# Timing chromosomal abnormalities using mutation data

**DOI:** 10.1186/gb-2011-12-s1-p39

**Published:** 2011-09-19

**Authors:** Steffen Durinck, Christine Ho, Nicholas J Wang, Wilson Liao, Lakshmi R Jakkula, Eric A Collisson, Jennifer Pons, Sai-Wing Chan, Ernest T Lam, Catherine Chu, Kyunghee Park, Sung-woo Hong, Joe S Hur, Nam Huh, Isaac M Neuhaus, Siegrid S Yu, Roy C Grekin, Theodora M Mauro, James E Cleaver, Pui-Yan Kwok, Philip E LeBoit, Gad Getz, Kristian Cibulskis, Jon C Aster, Haiyan Huang, Elizabeth Purdom, Jian Li, Lars Bolund, Sarah T Arron, Joe W Gray, Paul T Spellman, Raymond J Cho

**Affiliations:** 1 Life Sciences Division, Lawrence Berkeley National Laboratories, CA, USA; 2 Department of Statistics, University of California, Berkeley, CA, USA; 3 Department of Dermatology, University of California, San Francisco, CA, USA; 4 Emerging Technology Research Center, Samsung Advanced Institute of Technology, Korea; 5 Samsung Electronics Headquarters, Seoul, Korea; 6 San Francisco Dermatopathology Service, San Francisco, CA, USA; 7 The Eli and Edythe L Broad Institute of Harvard and MIT, Cambridge, MA, USA; 8 Department of Pathology, Brigham and Women’s Hospital, Boston, MA, USA; 9 Beijing Genomics Institute-Shenzhen, Shenzhen, China; 10 Institute of Human Genetics, Aarhus University, Aarhus, Denmark; 11 Biomedical Engineering Department, Oregon Health and Science University, Portland, OR, USA

## Background

Tumors accumulate large numbers of mutations and other chromosomal abnormalities because of a breakdown in genomic repair mechanisms, which is a hallmark of tumors. Not all of these abnormalities are thought to be crucial for tumor growth and progression, and it is a question of great importance to try to identify critical abnormalities, particularly as possible targets for treatment. A strong indicator of the importance of an abnormality is the order in which it occurred relative to other abnormalities, with triggering events likely to have occurred earlier.

## Methods

In general, we cannot directly observe the temporal progression of a tumor; however, for some types of chromosomal gains and losses, the mutations within the event can be classified as having occurred before or after the event by virtue of being homozygous or heterozygous. The simplest case is copy-neutral loss of heterozygosity (CN-LOH), in which it is reasonable to assume that homozygous mutations occurred before the LOH event and that heterozygous mutations occurred after the LOH event. Using sequencing data, we developed a probabilistic model for the observed allele frequency of a mutation, which allows us to estimate the true proportion of pre- and post-event mutations. Specifically, we modeled the number of reads with the mutation as a mixture model of binomials and estimated the mixing proportion. On the basis of this model, we can estimate this proportion for all LOH events within a sample and give a temporal ordering to the events within a sample. We applied this method to exome capture sequencing data that were obtained from eight primary cutaneous squamous cell tumors and matched normal pairs [1].

## Results

An immediate novel result of the analysis was that CN-LOH of chromosome 17p was temporally ordered as the first event (among CN-LOH events) in all four of the eight tumors that had CN-LOH of this region. The well-known tumor suppressor gene *TP53* is located in the CN-LOH region and has pre-CN-LOH mutations in all of the samples, further strengthening the role of *TP53* as a trigger for tumor progression.

## Conclusions

Our method gives novel insight into the biology of tumor progression through a quantitative evaluation of temporal ordering of chromosomal abnormalities. Moreover, it yields a quantitative measure for comparing samples to highlight driver mutations and events.

## References

[B1] Durinck S Ho C Wang N Liao W Jakkula LR Collisson EA Pons J Chan SW Lam ET Chu C Park K Hong SW Hur JS Huh N Neuhaus IM Yu SS Grekin RC Mauro TM Cleaver JE Kwok PY LeBoit PE Getz G Cibulskis K Aster JC Huang H Purdom E Li J Bolund L Arron ST Gray JW Temporal dissection of tumorigenesis in primary cancers Cancer Discov 2011 1 OF1 OF7 10.1158/2159-8290.CD-11-0028 PMC3187561 21984974

